# Surgical treatment of a patient with live intracranial sparganosis for 17 years

**DOI:** 10.1186/s12879-022-07293-7

**Published:** 2022-04-09

**Authors:** Jialing Hu, Kaili Liao, Xiaojin Feng, Danling Jiang, Hailin Liu, Qingcui Zheng, Hai Qiu, Fuzhou Hua, Guohai Xu, Chunhua Xu

**Affiliations:** 1 Department of Emergency Medicine, The Second Affiliated Hospital of Nanchang University, Nanchang, 330006 Jiangxi China; 2 Department of Clinical Laboratory, The Second Affiliated Hospital of Nanchang University, Nanchang, 330006 Jiangxi China; 3 Department of Anesthesiology, The Second Affiliated Hospital of Nanchang University, Nanchang, 330006 Jiangxi China; 4 Department of Neurosurgery, The First Affiliated Hospital of Nanchang University, Nanchang, 330006 Jiangxi China; 5 Department of Urology, XingGuo People’s Hospital, Ganzhou, 341000 Jiangxi China

**Keywords:** Sparganosis mansoni, Intracranial infection, Parasitic disease, Neuronavigation, Case report

## Abstract

**Background:**

The incidence of sparganosis, especially intracranial live sparganosis is very low in China. Due to the lack of typical clinical manifestations, it is difficult to make a clear preoperative diagnosis of the disease, which often leads to delays the disease and serious consequences.

**Case presentation:**

A 23-year-old man presented with a 17-year history of intermittent seizures and right extremity numbness and weakness. Magnetic resonance imaging (MRI) showed patchy, nodular and line-like enhancement. Enzyme-linked immunosorbent assay (ELISA) detected positive antibodies to Spirometra mansoni in peripheral blood and cerebrospinal fluid (CSF). In addition, during the operation, an ivory-colored live sparganosis was removed under the precise positioning of neuronavigation, and the patient was diagnosed with cerebral sparganosis. The patient began praziquantel and sodium valproate treatment after the operation, and was followed up for 3 months. There was no recurrence of epilepsy, and the weakness and numbness of the right limb improved.

**Conclusion:**

Nonspecific clinical manifestations often make the diagnosis of cerebral sparganosis difficult, and a comprehensive diagnosis should be made based on epidemiological history, clinical manifestations, ELISA results and imaging findings. Surgery is the preferred method for the treatment of cerebral sparganosis, and more satisfactory results can be achieved under the precise positioning of neuronavigation.

**Supplementary Information:**

The online version contains supplementary material available at 10.1186/s12879-022-07293-7.

## Background

Human sparganosis is a rare parasitic disease caused by the larvae of the genus Sparganum, first described by Manson in 1882 [1]. Sparganosis infection has been reported sporadically all over the world, but it is most common in Southeast Asia, including countries such as China, Japan and South Korea [2]. The main transmission routes of sparganum include eating raw fish fillets, frogs, snake gall or partially cooked frog meat and snake meat; or sticking frog meat on wounds to promote wound healing, and drinking raw water. Sparganum usually invades subcutaneous tissue and muscle, but rarely affects the brain [3]. The common symptoms of sparganosis are seizures, headaches, hemiplegia and sensory disturbances, depending on the location of the brain disease [4, 5]. Studies have shown that sparganosis can survive in the human body for up to 20 years [6, 7], but as far as we know, there have been very few patients with sparganosis mansoni in whom sparganosis survived in the brain for a long time. Here, we report an interesting case of sparganosis infection, which may have persisted intracranially for 17 years. The patient was treated with praziquantel and anti-epileptics, and the craniotomy was successfully performed under neuronavigation after the treatment failed. In addition, we reviewed the literature on the disease.

## Case presentation

A 23-year-old man presented to the neurosurgery department on August 21, 2020, after suffering intermittent seizures and numbness and weakness of the right limb for 17 years. Before that, the family members reported that the patient had a limb convulsion at the age of 6, the right limb was numb and weak, and the symptoms were not considered. On June 14, 2016, due to another epileptic seizure, serum and cerebrospinal fluid were collected and tested at the Jiangxi Provincial Institute of Parasitic Disease Control and IgG antibody testing. The test results showed that both serum and cerebrospinal fluid were positive for Spirometra mansoni antibodies (Table 1); cerebrospinal fluid acid-fast staining and ink staining were normal; the Paneth test result was positive; the total number of white blood cells was 0.01*10^9^/L; and the cerebrospinal fluid protein, sugar, and chloride were normal. The patient had no history of trauma, surgery, genetic or other chronic medical conditions, and did not complain of pain, itching, or other symptoms associated with the lesion. After further careful questioning, it was revealed that the patient was born in a rural area. When he was young, he often played in paddy fields, had contact with frogs, ingested undercooked frog meat, and had a habit of drinking mountain spring water. The patient refused surgery. Praziquantel was administered at a dose of 20 mg/kg three times a day for 15 consecutive days. The patient’s symptoms did not improve significantly. On August 18, 2020, the serum Spirometra mansoni IgG antibody test was performed again, and the results were not negative for Spirometra mansoni antibodies (Table 1).

**Table 1:** Laboratory examination of sparganosis mansoni

Date	Term (ELISA)	Result	Reference value
June/2016	Sparganosis mansoni (IgG antibody)		
	1:100	+	–
	1:200	+	–
	1:400	+	–
	1:800	–	–
	1:1600	–	–
	1:3200	–	–
August/2016	Sparganosis mansoni (IgG antibody)		
	1:100	+	–
	1:200	+	–
	1:400	+	–
	1:800	+	–
	1:1600	+	–
	1:3200	–	–
April/2016	Parasites		
	Paragonimiasis (antibody)	–	–
	Schistosomiasis (antibody)	–	–
	Sparganosis mansoni (antibody)	+	–
	Echinococcosis (antibody)	–	–
	Angiostrongylus cantonensis (antibody)	–	–
	Cysticercosis (antibody)	–	–
	Toxoplasma gondii IgG (parasite)	–	–
	Toxoplasma gondii IgM (parasite)	–	–

–: Negative, + : positive

**Fig. 1:**
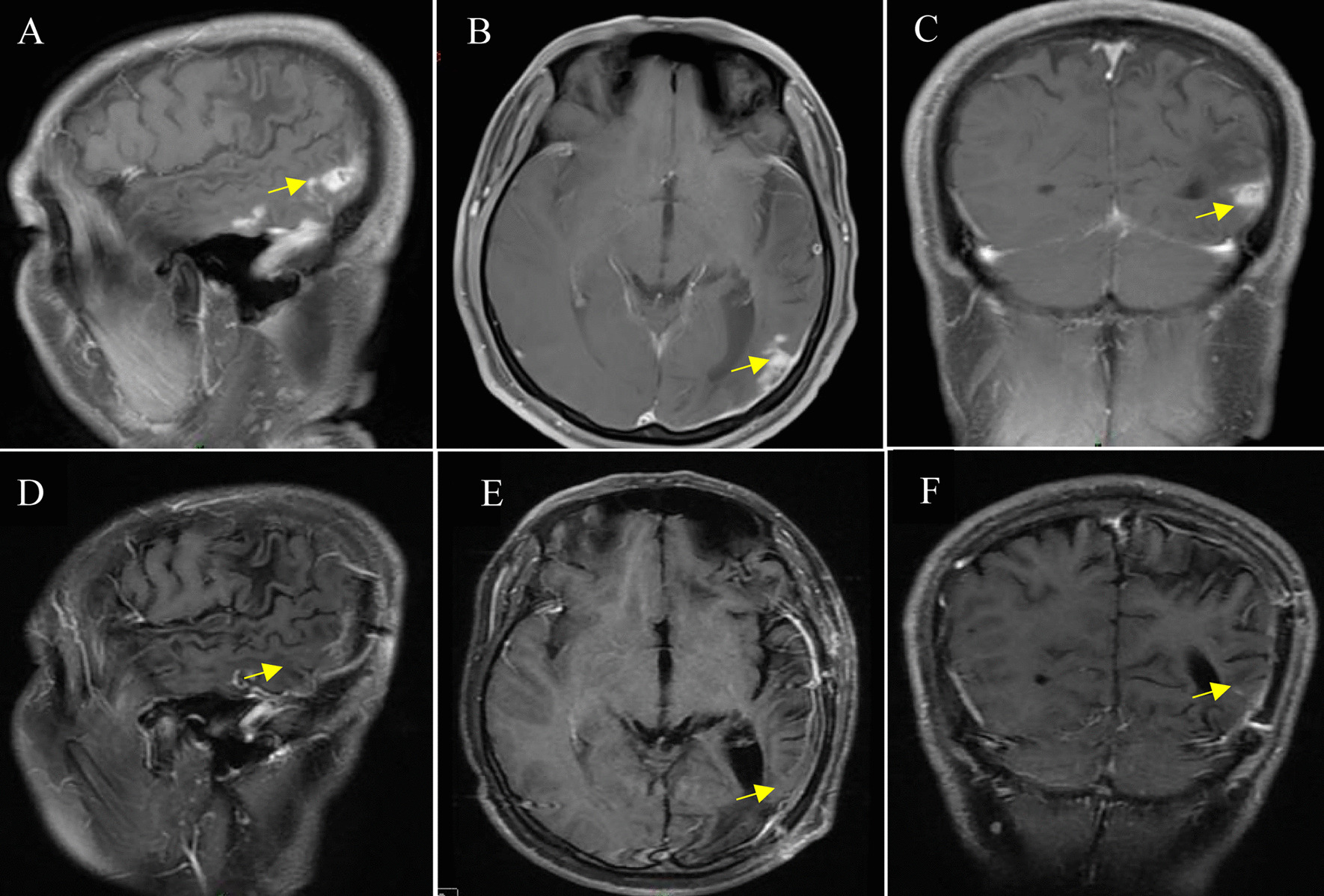
Enhanced MRI of the brain. Preoperative sagittal (**A**), axial (**B**), and coronal (**C**) T1-enhanced MR images show Patchy, nodular and linear enhancements can be seen in the brain parenchyma and adjacent meninges of the left temporoparietal occipital lobe. Postoperative MR images show gross total resection of the Parasite (**D**–**F**)

The patient had hypoesthesia in the right limb, and the remaining nerve function was normal. Laboratory tests including complete blood count, biochemical markers, urinalysis and routine stool tests were within normal limits, and no eosinophilia was observed at 0.12*10^9^/L (reference value range was 0.02–0.52*10^9^/L). The patient’s results evaluating peripheral blood paragonimiasis, schistosomiasis, hydatid disease, Angiostrongylus cantonensis, cysticercosis antibody negative and Toxoplasma gondii IgG and IgM were negative (Table 1). On August 25, 2020, enhanced MRI of the brain showed that the left temporoparietal occipital lobe brain parenchyma and adjacent meninges showed obvious patchy, nodular and line-like enhancement; a low diffusion-weighted imaging (DWI) signal, and a slightly high apparent diffusion coefficient (ADC). We initially considered the possibility of neoplastic lesions. (Fig. 1A–C). Based on further clarification of the diagnosis, a craniotomy was performed on August 25, 2020, to remove the lesion (Fig. 1D–F).

## Operation and pathological findings

**Fig. 2:**
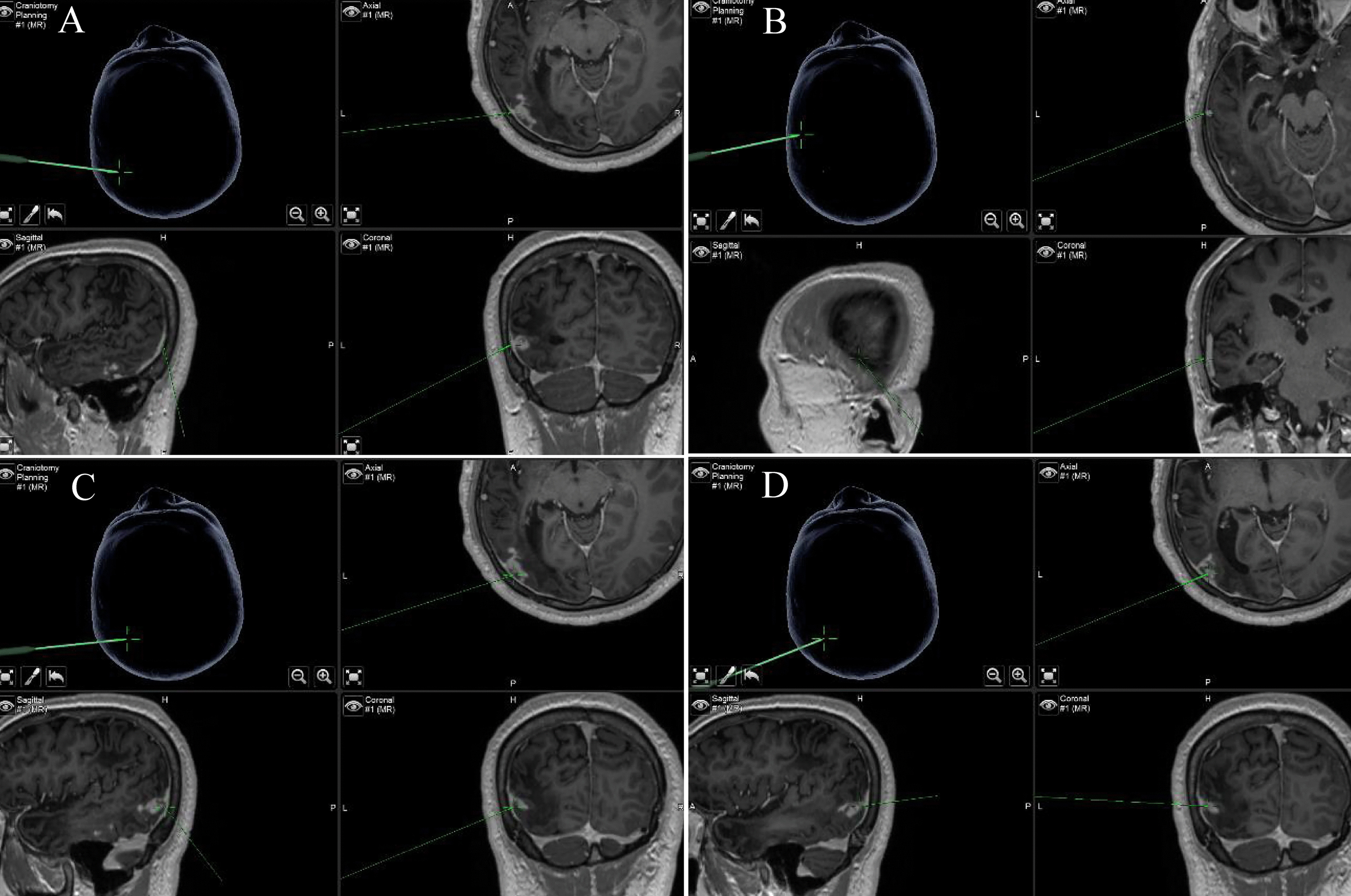
Neuronavigation and localization. (**A**–**D**) Neuronavigation combined with contrast-enhanced MRI to pinpoint lesions from sagittal, axial, and coronal, as indicated by green arrows

After successful general anesthesia, the surgical area of the patient was disinfected and draped, a left temporoparietal horseshoe-shaped incision 4–5 cm long was made, and the bone flap with a diameter of approximately 3 cm was removed with a milling cutter. MRI neuronavigation was used to locate multiple enhancement lesions in the temporal region (Fig. 2A–D), bordering the posterior border of the posterior lesions. Electrocoagulation incised the cortex to perform resection of the posterior lesions of the left temporal lobe. A live ivory-white parasite was removed from the surface of the lesions. The parasite was approximately 12 cm long and 0.5 cm wide, the head end was enlarged, the body was slender, and it moved actively like an amoeba in normal saline. The surgical area was then separated along the lesion, and a grayish-yellow, tough-textured area of edema around the lesion with edema was seen, and subtotal resection was performed. Suspicious parasites were found at the bottom of the inferior temporal gyrus with obvious enhancement, which were surrounded by granulomas, and the granulomas were excised. The lesions were taken for the anterior and posterior parts of the temporal lobe and sent for biopsy. Care was taken to avoid breaking the larvae and leaving any larval residue during the procedure. For illustrative purposes, we have also attached a video clip of the surgical procedure (Additional file 1: Video 1).

**Fig. 3:**
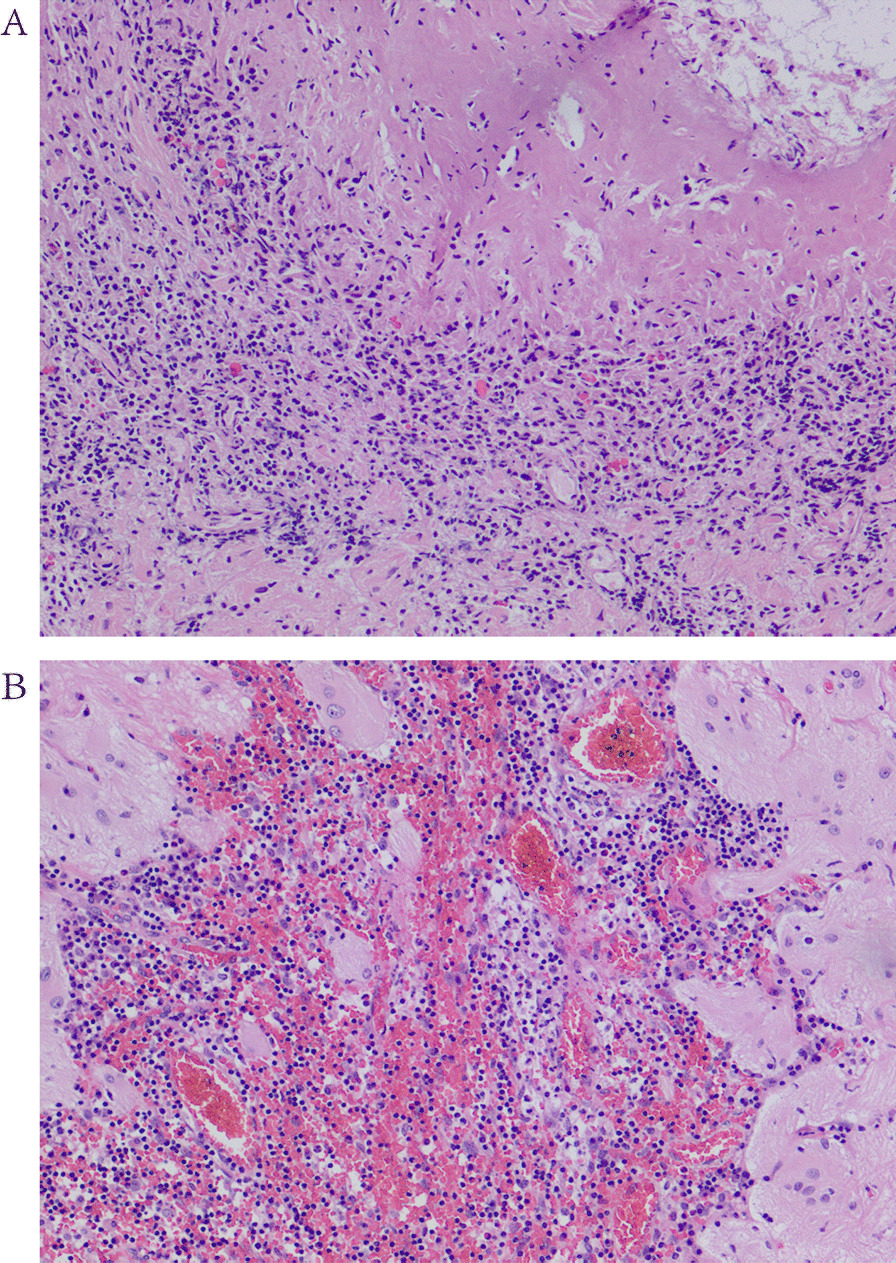
Hematoxylin–eosin staining method displayed under a microscope at 200 time. (**A**, **B**) Brain tissue edema, focal microcystic degeneration, glial cell proliferation in some areas, infiltration of a large number of lymphocytes, plasma cells and eosinophils, necrosis in some areas, surrounding tissue cells and multinucleated giant cells, and a large number of new small blood vessels dilated, Hyperemia, a large number of lymphocytes around the blood vessels infiltrate and form a sleeve-like structure

Pathological examination: The size of the resected tissue was 26 × 20 × 9 mm. The specimen was gray–white, and the cut surface was soft. According to the microscopic examination results of the Department of Pathology of Nanchang University (Jiangxi Province), brain tissue edema, focal microcystic degeneration, gliosis in some areas, a large number of infiltrated lymphocytes, plasma cells and eosinophils, necrosis in some areas, peripheral necrosis surrounded by histiocytes and multinucleated giant cells were observed. In addition, a large number of new small blood vessels were dilated and congested, and a large number of lymphocytes infiltrated around the blood vessels to form a cuff-like structure (Fig. 3A, B). The patient recovered smoothly after surgery, and was instructed to take 500 mg of sodium valproate sustained-release tablets daily. After 3 months, the drug was discontinued without seizures. After surgery, the weakness and numbness of the right limb improved, and there was no neurological deficit. The patient was then discharge from the hospital.

## Discussion and conclusions

Sparganosis is a chronic parasitic disease caused by the larva of Spirometra mansoni, which has not attracted great attention due to its rarity. Here, we report a case of sparganosis that has survived for a long time in the brain. Sparganosis is not common in brain parasitic infections. How sparganum enters the brain remains unclear. Based on previous studies, the following pathways are proposed. When a patient is infected, the larvae enter the abdominal cavity through the digestive tract, then they migrate further to the diaphragm and mediastinum, reach the neck, and finally enter the brain through the foramen magnum [8]. After drinking raw water, the protocercaria enters the intestinal vein blood circulation with the puncture gland of the tail, and then enters the skull via blood flow. The proteolytic enzymes in the cephalic segment enable Schizomeraria to hydrolyze proteins and peptides, thus promoting the penetration of thick tissues and membranes [9, 10]. The most common symptoms of sparganosis include seizures, headaches, hemiplegia, and sensory disturbances. Different symptoms depend on the location of the disease [9, 10]; that is, different symptoms vary according to the location of the sparganosis in the skull. Our patient had intermittent seizures with recurrent weakness in the right extremity. Clinically, sparganosis is often misdiagnosed due to atypical features, irregular intracranial localization, and atypical epidemiology, and is often misdiagnosed as glioma.

**Fig. 4:**
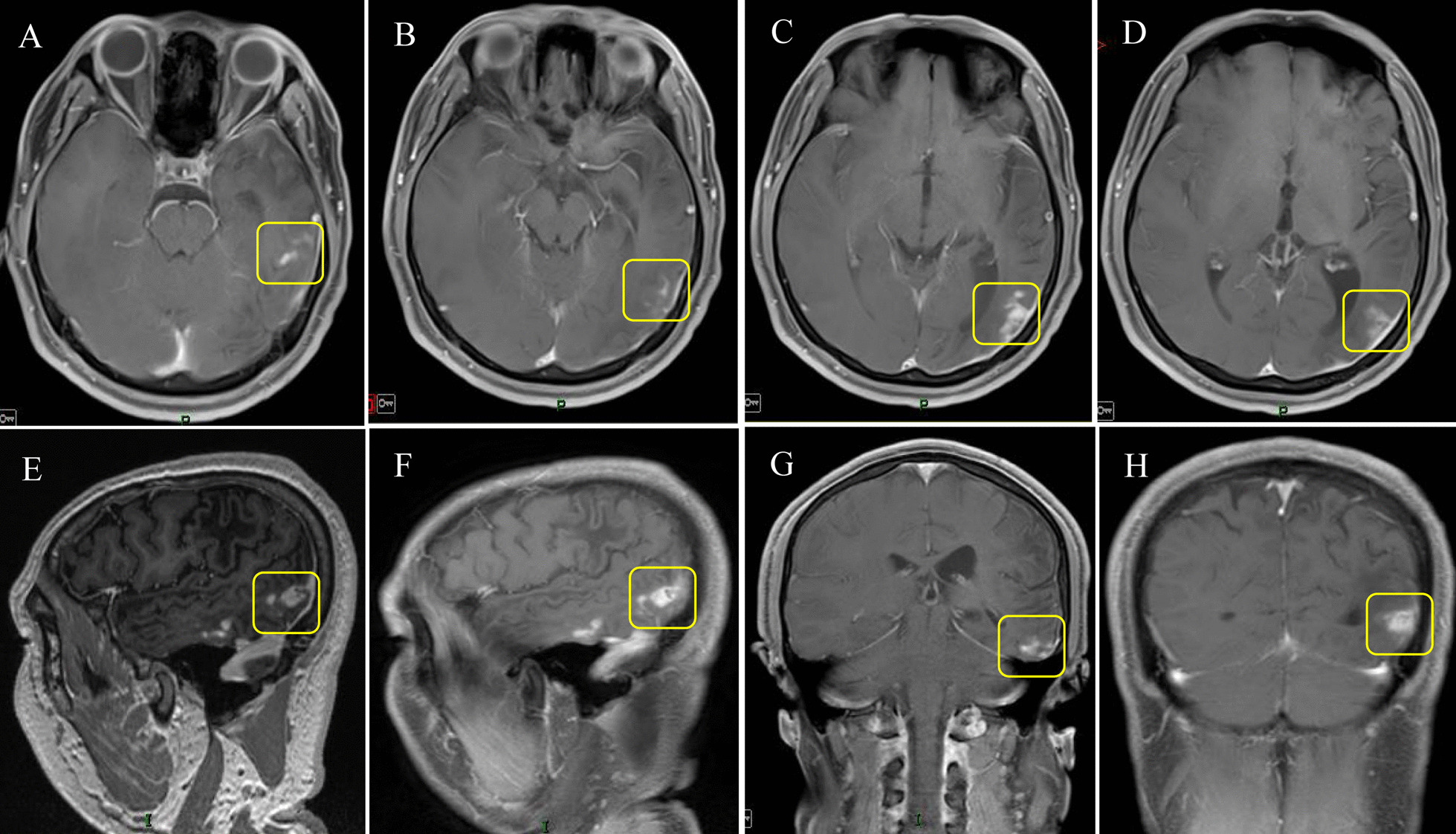
Scar tissue hyperplasia on contrast-enhanced MRI. **A**–**H** Contrast-enhanced MRI shows hyperplasia of scar tissue produced by the long-term parasitism of Sparganus, as shown in the yellow box

According to the characteristics of sparganum, which causes obvious symptoms, the lifespan of sparganum is usually less than one year. However, according to the patient's statement, the present sparganum was thought to have lived in his skull for 17 years. Long-term survival of the parasite is possible due to the ability of the parasite to alter the host's immune response [11]. In our case, contrast-enhanced MRI showed hyperplasia of scar tissue (Fig. 4A–H), and intraoperative granulomatous lesions were seen. In addition, pathological examination showed gliosis, and a large infiltration of lymphocytes, plasma cells, and eosinophils also suggested this. This outcome is a chronic pathological process. Studies have shown that the immature immune system and blood–brain barrier in children and young adults can allow larvae to enter the brain more easily, thereby making them more prone to sporocystosis [8, 12, 13] and longer parasite survival times in humans.

**Fig. 5:**
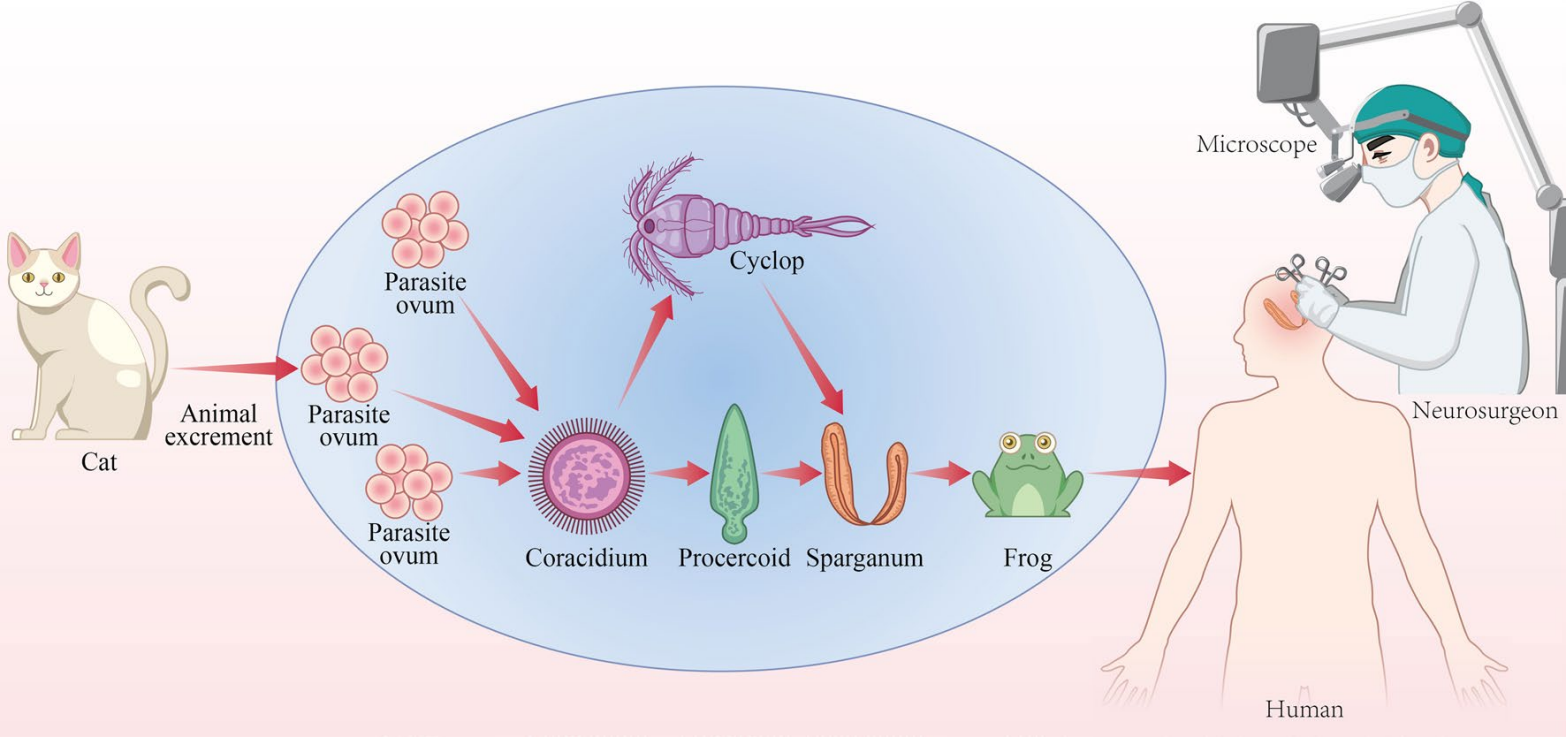
Possible infection routes of sparganosis. Sparganosis parasites in the small intestines of cats, dogs, etc. The eggs are excreted with the feces and hatched in the water. After being swallowed by the first intermediate host, the larvae are shed in the body and become protocercaria. The protocercaria containing the protocercaria are swallowed by the second intermediate host tadpoles and developed into Sparganosis, when tadpoles develop into frogs, they migrate to the thighs and calf muscles of the frog to parasitize. The skin may be damaged during playing in the ridge. After contact with frogs, the sparganosis larvae enter the blood through the skin break and enter the skull with the blood flow. Produce symptoms of limb numbness and fatigue (Fig. 3 is our own drawing through Adobe Illustrator CS5 software, the picture is an original work)

Children often play in pools or streams, which may increase exposure to parasite-contaminated water [14]. Our patient liked to play in ridges as a child, had a history of contact with frogs and had a habit of drinking local mountain water and eating undercooked frogs that may have contributed to the infection. We speculate that the possible route of infection is: sparganosis parasites in the small intestines of cats, dogs, etc. The eggs are excreted with the feces and hatch in the water. After being swallowed by the first intermediate host, the larvae are shed in the body and become protocercaria. The protocercaria are swallowed by the second intermediate host tadpoles and develop into sparganosis; when tadpoles develop into frogs, the organisms migrate to the thighs and calf muscles of the frog to parasitize. The skin may be damaged during playing in the ridge. After contact with frogs, the sparganosis larvae enter the blood through a break in the skin, or by eating undercooked frog meat containing Schizomeraria which then enter into the skull with the blood flow, resulting in symptoms of epilepsy and limb numbness and weakness (Fig. 5). Considering that many people in rural areas like to drink mountain spring water, local water sources should also be investigated. Therefore, a comprehensive analysis and diagnosis, detailed medical history collection is very important in the clinical diagnosis process, especially when the route of transmission is not clear.

In addition to a reliable epidemiological history, the diagnosis of cerebral sparganosis is closely related to the lesion site [15]. The lesions are mainly located in the parietal lobe, frontal lobe, occipital lobe, cerebral ganglia and basal ganglia. Neuroimaging also plays an important role in the diagnosis of sparganosis. The main feature on MRI is the tunnel sign seen on the sequence after comparison; because live worms migrate in a wave motion. The second characteristic is an aggregated ring- or bead-like enhancement, which represents inflammatory granuloma. Finally, due to the long course of the disease, alternate changes in different stages in the same image are common. In our patients, head MRI showed patchy, nodular and linear enhancement. The possibility of neoplastic lesions has been considered, so familiarity with the imaging findings of cerebral sparganosis is crucial for its diagnosis.

Cerebral sparganosis is a rare zoonotic infectious disease. The differential diagnosis of cerebral sparganosis is usually exclusive, including brain tumors and inflammatory granuloma of various causes, such as fungal diseases, tuberculosis or other parasitic infections [16, 17]. The corresponding tumor markers in the brain tumor tests were positive, and the blood supply of enhanced magnetic resonance imaging was significantly enhanced. Inflammatory granuloma may be round enhancement with or without peripheral edema, but atrophy or expansion of brain tissue adjacent to the ventricle is usually absent [16]. Tuberculosis usually presents as symptoms of combined poisoning, which can be distinguished by a positive antibody to mycobacterium tuberculosis. Some studies have shown that, regardless of the clinical outcome, there is no direct relationship between the absolute count of peripheral blood eosinophils and the clinical outcome, and its absolute count cannot be used as an effective prognostic indicator [14]. Similar results were obtained in our case, and no increase in absolute eosinophil counts was observed.

In recent years, the incidence rate of foodborne parasitic diseases such as mansoni disease has increased, which is a continuous concern for food safety and community health. At present, a growing number of doctors suggest that surgery is the best way to treat sparganosis mansoni [15]. Removal of the entire worm, especially the first segment, is essential for the patient to obtain a radical cure. It is important to consider that surgery is necessary only in patients with live parasites [13, 18]. Therefore, it is necessary to determine the status of the parasite before resection. The changes in the location of the lesion or the deterioration of the patient’s symptoms on imaging are indicators of the survival of the parasite. However, when the parasite cannot be removed surgically, such as when the patients has multiple organ infections, praziquantel can be used for medical treatment. Recent studies have shown that high-dose prolonged (daily praziquantel dose of 75 mg/kg body weight, the drug is administered in three divided 10-day courses) praziquantel treatment is effective in patients with cerebral sparganosis [14, 19]. A 7- to 10-fold increase in drug concentration in the cerebrospinal fluid or brain parenchyma is required to effectively kill the parasite [20, 21]. This is also a possible reason why the conventional dose of 20 mg/kg/day was less effective in our case. Of note, follow-up examinations should be performed regularly to ensure that no larvae remain in the brain after surgery. In addition, many studies have shown that serum and cerebrospinal fluid-ELISA has high sensitivity [12, 15, 22] and specificity [23] for the diagnosis of cerebral sporocystosis.

In this study, we combined epidemiological history, MRI data, ELISA detection of parasite antibodies, and surgical treatment to diagnose a patient with intracranial sparganosis with epileptic seizures and limb numbness [22]. According to the medical history, MRI manifestations and pathological results, it is considered that intracranial survival can persist up to 17 years, and the parasites can be successfully removed by using the advantages of precise positioning of neuronavigation [24], and good postoperative recovery. In clinical practice, it is misdiagnosed due to atypical signs and symptoms of cerebral sporocystosis and limited laboratory testing methods. Here, we report a real picture of the disease, and we hope that our findings will improve the understanding of cerebral sporocystosis and provide new insights into the treatment and management of the disease, leading to more favorable treatment options.

Finally, we acknowledge some limitations of this study. First, the short follow-up time in this study makes it necessary to continue long-term MRI follow-up studies after clinical symptoms have improved. Second, since this study is retrospective, it may introduce bias into the findings. Third, due to the limitations of morphological examination, molecular techniques such as next-generation sequencing combined with whole-genome amplification appear to be directly applicable to the molecular diagnosis of parasites [25, 26]. Therefore, DNA sequencing is crucial for reliable species identification.

To the best of our knowledge, intracranial sparganosis with a survival time of 17 years has never been reported. Surgical resection is generally considered to be the best treatment for sparganosis. This case shows that the variability of clinical manifestations of intracranial sparganosis may lead to delayed diagnosis or missed diagnosis of sparganosis. It is necessary to guide the diagnosis and differential diagnosis of this group of patients by combining the epidemiological history, clinical manifestations, parasite antibody detection, head radiology, pathological biopsy and parasite identification. In addition, relevant publicity work should be done to prevent people from eating raw frogs, fish and snakes, to prevent the disease from spreading in endemic and nonendemic areas.

## Supplementary Information

**Additional file 1: Video S1.** A live parasite grabbed from the surface of the lesion, freely and completely pulled out, about 12 cm in length, 0.5 cm in width, enlarged head, slender body, and nodular peristalsis.

## Data Availability

All data generated or analysed during this study are included in this published article.

## Abbreviations

MRI: Magnetic resonance imaging
ELISA: Enzyme-linked immunosorbent assay
CSF: Cerebrospinal fluid
DWI: Diffusion weighted imaging
ADC: Apparent diffusion coefficient
